# Computed tomographic development of physeal osteochondrosis in pigs

**DOI:** 10.1186/s12917-019-2163-7

**Published:** 2019-12-17

**Authors:** Kristin Olstad, Bjørn Wormstrand, Jørgen Kongsro, Eli Grindflek

**Affiliations:** 1 Faculty of Veterinary Medicine, Department of Companion Animal Clinical Sciences, Equine Section, Norwegian University of Life Sciences, Post Box 369 Sentrum, 0102 Oslo, Norway; 2 Norsvin SA, Storhamargata 44, 2317 Hamar, Norway

**Keywords:** Growth plate, Helical computed tomography, Limb deformities, Longitudinal studies, Osteochondrosis, Physis, Swine, Vascular failure

## Abstract

**Background:**

Articular osteochondrosis follows a dynamic development pattern. Lesions arise, in incidence peaks compatible with failure of cartilage canal vessels during incorporation into bone, and can also resolve. Lesions that resolve before examination at a single time point will constitute false-negative diagnoses. The aim of the study was to identify physeal osteochondrosis lesions in pigs and monitor their development by computed tomography (CT), to determine if they follow a similar dynamic development pattern to articular osteochondrosis.

**Results:**

Thirteen physes were evaluated bilaterally in up to eight biweekly CT scans from 18 male Landrace pigs age 70–180 days (total: 112 scans), generating 2912 scores. There were 1754 (60%) lesion-negative scores and 1158 (40%) lesion-positive scores. Positive scores comprised 138 lesions present at the start and 235 lesions that developed during the study, from 4 to 32 lesions per physis (median: 15 lesions). There were 1–2 peaks in the incidence curves for 12/13 examined physes, the exception being the proximal humerus. Positive scores also included 785 times that lesions persisted, from 1.3–4.8 examination intervals per lesion (median: 2.8 intervals). Negative scores included 190 times that lesions resolved, from 19 to 100% of lesions per physis (median: 65%). Lesions resolved by filling with bone from marginal sclerosis and reparative ossification centres. In the distal scapula and distal fibula, perichondrial new bone formation occurred that led to permanent enlargement of physeal regions. Angular limb deformity was not identified in any pig.

**Conclusions:**

Physeal osteochondrosis followed a similar dynamic development pattern to articular osteochondrosis. There were peaks in the incidence curves, compatible with failure of vessels during incorporation into bone. In some physes, osteochondrosis led to permanent enlargement, potentially relevant for decubital ulcers. The relationship between physeal osteochondrosis and angular limb deformity must be examined further in pigs over 6 months old in future.

## Background

Osteochondrosis is the most common developmental orthopaedic disease in pigs [1, 2]. It can occur in both the sub-articular epiphyseal growth cartilage and the metaphyseal growth plate or physis [3]. Epiphyseal growth cartilage has a blood supply that is organised as anatomical end arteries that run inside cartilage canals [4, 5]. The mid-portion of canals is incorporated into the ossification front at the periphery of the epiphysis as the individual matures [4, 5]. Failure of the blood supply during incorporation leads to ischaemic chondronecrosis axial to the point of incorporation, around the distal portion of the end artery and at mid-depth of the growth cartilage, known as osteochondrosis latens [4, 5]. Continued ossification around, but not within the lesion leads to focal delay in endochondral ossification, or osteochondrosis manifesta [4–7]. Articular osteochondrosis lesions can resolve or progress to pathologic fracture and OCD fragments in joints, associated with effusion, pain and lameness/leg weakness [1, 7, 8]. A recent cross-sectional study indicates that the pathogenesis of physeal osteochondrosis is similar to the pathogenesis of articular osteochondrosis [9]. Physeal lesions were also compatible with failure of end arteries at the point of incorporation into bone [9]. Devascularisation was associated with retention of viable hypertrophic chondrocytes, rather than necrosis, and focal delay in endochondral ossification/osteochondrosis [3, 9–11]. The start of lesions: failure of vessels at the point of incorporation, and the effect: focal delay in ossification, were therefore the same for both physeal [9] and articular osteochondrosis [4, 5]. Physeal osteochondrosis has been associated with angular limb deformity [3]. Early, subclinical lesions were most common in 4 month-old pigs, whereas pronounced deviation centred at the lesion sites was most apparent in 6–8 month-old pigs [3]. Contemporary pigs reach slaughter weight by 5–6 months of age, thus angular limb deformity is unlikely to cause a problem in this population. However, it remains important to control physeal osteochondrosis as angular limb deformity would lead to uneven loading predisposing for claw and joint problems [3]; important reasons for premature culling of breeding sows [12]. Heritability (h^2^) has been estimated at 0.30 for articular osteochondrosis and 0.14 for physeal osteochondrosis, thus prevalence in both locations can be reduced through selective breeding [13].

In 2008, advanced computed tomography (CT) was introduced as a tool for routine phenotyping of boar candidates for selection to the breeding programme in Norway [2]. A whole-body scan is carried out at 120 kg live body weight for standardised quantification of lean meat and fat percentage [2]. Computed tomography is also ideal for evaluation of skeletal health, and in 2014, CT was histologically validated for diagnosis of articular osteochondrosis in the distal femur of pigs [2, 14]. This paved the way for the first true longitudinal monitoring of the development of early lesions in six major limb joints of pigs [8]. A dynamic development pattern of lesions arising and resolving spontaneously before certain joint-specific, upper age thresholds was known from longitudinal radiography of the fetlock, hock and stifle joints of horses [15, 16]. The longitudinal CT study of pigs revealed that there were 1–2 peaks in the incidence graphs for 6/6 examined joints, i.e. times when many lesions arose at the same predilection site in several different pigs [8]. It was not possible to identify any external trigger that was common to all pigs and could explain the peaks. Instead, it was hypothesised that there was an internal trigger, namely incorporation of vessels into bone, that was common to multiple pigs at a similar time [8]. The pig CT study therefore augmented the horse studies by confirming that there can be one or more waves of lesions developing up to the age thresholds for lesions arising [8, 15, 16]. The pig CT study also confirmed that from 51 to 69% of initiated articular lesions underwent resolution before 116 kg mean body weight [8]. Lesions that resolve before phenotyping at 120 kg body weight will constitute false-negative diagnoses [8]. It is important to know the development pattern of osteochondrosis, because it influences the accuracy of detection and, in the worst instance, can cause phenotypic selection to fail in reducing disease prevalence.

Osteochondrosis affects some physes more severely than others [3, 17]. If CT is to be used for phenotyping, it is necessary to confirm which physes are most severely affected in contemporary pigs. It must also be determined whether physeal osteochondrosis follows a similar pattern to articular osteochondrosis of lesions arising and resolving, with potential for false-negative diagnoses. In his cross-sectional study, Reiland (1978) detected lower osteochondrosis prevalence in the 15–18 month-old group of pigs and considered that lesions had healed, or that pigs with severe lesions had been culled [3]. Hill et al. (1984) found longitudinal radiography less accurate than radiography of slabs, and limited their conclusions to it being probable that lesions resolved [17]. In 1994, Bittegeko & Arnbjerg finally proved that lesions could resolve, but the report was limited to the distal ulnar physis [18]. The development pattern of physeal osteochondrosis must therefore be determined, and doing so by longitudinal CT may reveal valuable, new information about pathogenesis, as it did for articular lesions.

The aim of the current study was to identify physeal osteochondrosis lesions in pigs and monitor their development by CT, to determine if they follow a similar dynamic development pattern to articular osteochondrosis.

## Results

Angular limb deformity was not observed in any of the current examined pigs at any interval.

### Quantitative observations

Evaluation of 13 physes bilaterally in 112 scans generated 2912 lesion-positive and lesion-negative scores. By accident, the most caudad-positioned hind limb physis was outside the collimated window on eight occasions (0.3%). On four occasions, this occurred at the first interval and a diagnosis of lesion-negative was assumed, otherwise the diagnosis at the preceding interval was assumed. With the exception of a single, incomplete Type I Salter-Harris fracture in the left distal femoral physis of pig 18, all lesions were compatible with the definition of osteochondrosis given in the Methods section, below.

**Fig. 1:**
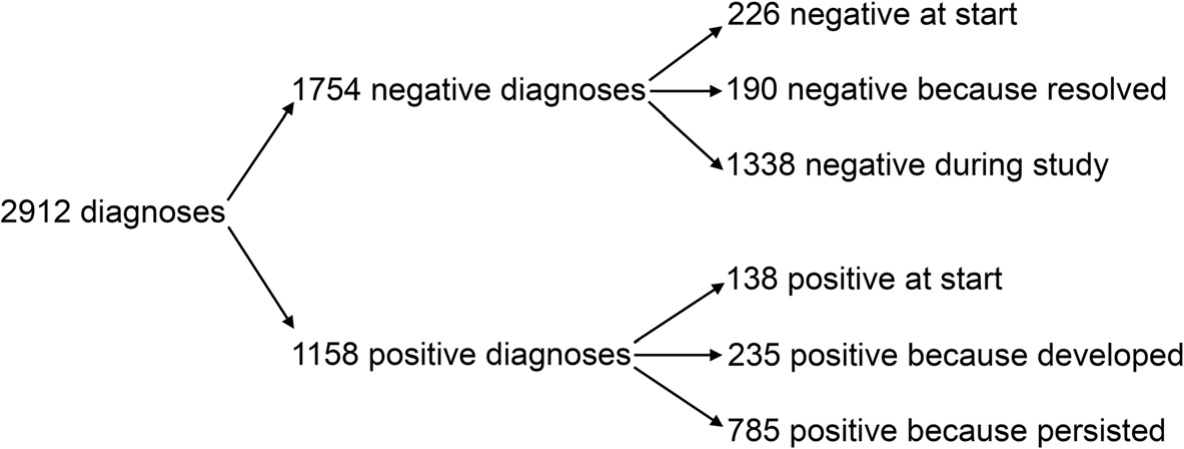
Flow chart of 2912 diagnoses

The 2912 diagnoses comprised 1754 (60%) lesion-negative scores and 1158 (40%) lesion-positive scores (Fig. 1). The negative scores were distributed as 226 physes that were negative at the start, 190 physes that were negative because a lesion from the preceding interval resolved and 1338 times that a physis was negative without having been positive at the preceding interval (Fig. 1). The positive scores included 138 lesions present at the study start, 235 lesions that developed during the study and 785 times that a lesion persisted from a preceding interval (Fig. 1). The three physes that were responsible for the most lesion-positive scores in the study were the distal femoral (*n* = 200), proximal humeral (*n* = 175) and distal ulnar (*n* = 154) physes (Table 1).

**Table 1:**
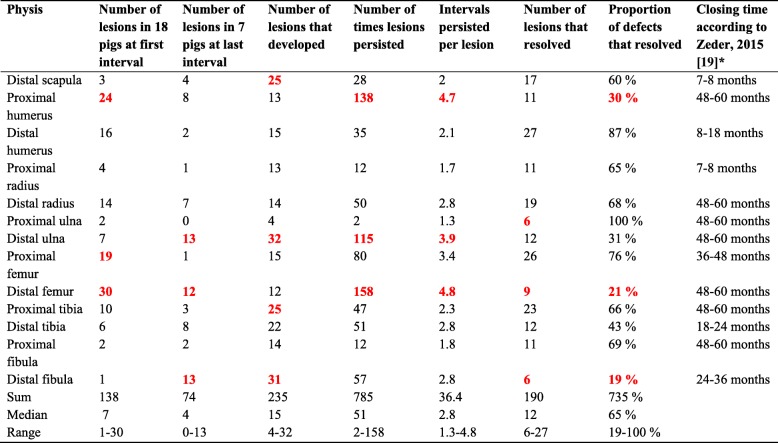
Scores per physis

Bold red text: The three poorest scores in column. *Reference chosen because it has the most comprehensive data and summarises eight other studies including Reiland, 1978 [20]

### Prevalence of lesions

At the study start and mean body weight 26 kg, the three most commonly affected physes were the distal femoral, proximal humeral and proximal femoral physes (Table 1). In the seven pigs that remained at the eighth interval and mean body weight 116 kg, lesions were most common in the distal ulnar, distal fibular and distal femoral physes (Table 1). Prevalence therefore remained relatively constant for some physes and varied slightly for other physes during the study. It was noted that although the proximal ulnar physis was lesion-negative at the eighth interval, it had previously been positive (Table 1). Prevalence was also different between proximal and distal physes within the same bone (Table 1). The distal physis was more commonly affected than the proximal physis in the radius, ulna, femur and fibula, whereas the reverse was true for the humerus and tibia.

### Incidence of lesions

**Fig. 2:**
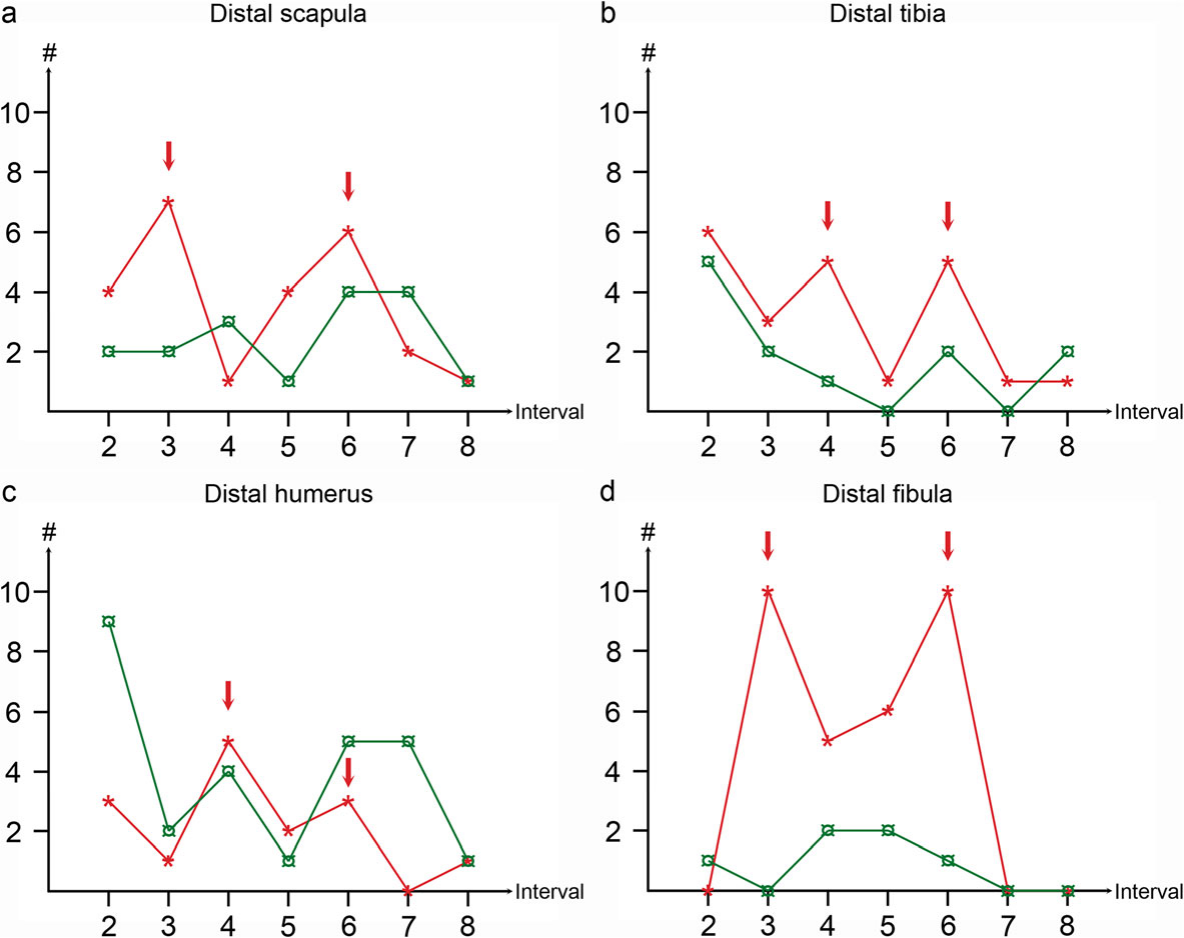
Graphs of lesions developing and resolving in selected physes. **a** Distal scapular physis. **b** Distal tibial physis. **c** Distal humeral physis. **d** Distal fibular physis. The graphs show the number of defects that developed (red asterisks, ) and resolved (green currency signs, ) on the y-axes, from the second to the eighth examination interval on the x-axes. In the four physes shown, incidence declined with two distinct peak values (red arrows). In one physis, incidence declined without any peaks, and in the remaining eight physes, incidence graphs declined with a single peak

The number of lesions that developed during the study was 235, ranging from 4 to 32 lesions per physis (median: 15 lesions; Table 1). Incidence declined with increasing age (Table 2). In the proximal ulnar and distal fibular physes, there were no new lesions at the seventh and eighth intervals, whereas in the proximal humeral, distal radial, distal ulnar and distal femoral physes, there were no new lesions at the eighth interval (Table 2). When plotted graphically, incidence declined evenly in the proximal humeral physis, and declined with two distinct peak values in the distal scapular, distal humeral, distal tibial and distal fibular physes (Fig. 2). In the remaining eight physes, incidence graphs declined with a single peak.

**Table 2:**
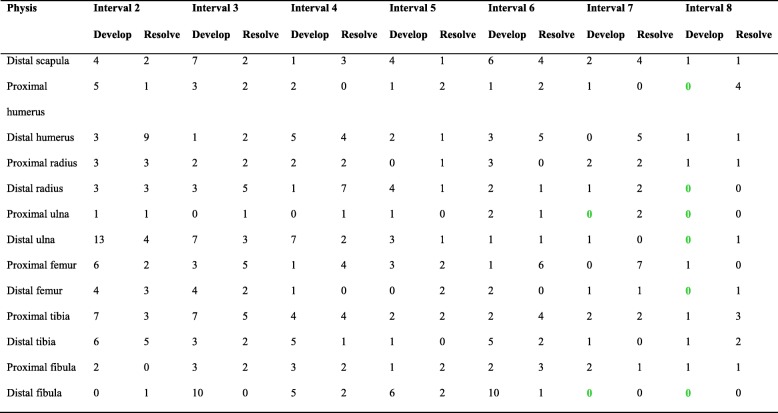
Incidence of lesions developing and resolving per physis during intervals 2–8

Bold green text: Zero new lesions developing at intervals 7 and 8

### Persistence of lesions

Persistence was examined by adding the number of lesions to the number of times lesions persisted, and dividing this by the number of lesions to generate an average for intervals persisted per lesion in each physis (Table 1). The range was from 1.3–4.8 intervals/lesion (median: 2.8 intervals/lesion), i.e. from 18 to 67 days. The three physes with the longest duration lesions were the distal femoral, proximal humeral and distal ulnar physes.

### Resolution of lesions

The number of defects that resolved was divided by the total number of lesions to determine the proportion of lesions that resolved per physis (Table 1). The range was from 19 to 100% of lesions (median: 65%). The three physes with the lowest proportion of lesions resolving were the distal fibular, distal femoral and proximal humeral physes. Three physes therefore stood out quantitatively as scoring badly in terms of high prevalence/incidence, long persistence and low proportion of lesions resolving during the study: the distal femoral, distal ulnar and proximal humeral physes. A fourth physis stood out because it had the lowest proportion of lesions resolving, namely the distal fibular physis.

### Lesions per pig and relationship to articular osteochondrosis

The distribution of the 2912 scores across the 18 pigs is shown in Table 3. In the same way as there were good and bad physes, there were good and bad pigs. When tested, there was no statistically significant correlation between the number of physeal and articular lesions (Table 3) on the study end date in the current pigs. When limited to the information available at 116 kg mean body weight, pigs 18, 13 and 15 were best for articular osteochondrosis, but pig 15 was worst for physeal osteochondrosis.

**Table 3:**
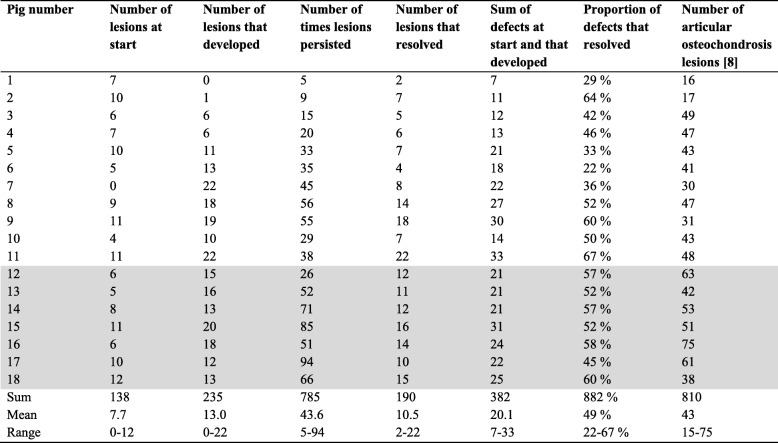
Scores per pig

Grey shading: The seven pigs that completed all eight intervals

### Qualitative observations

**Fig. 3:**
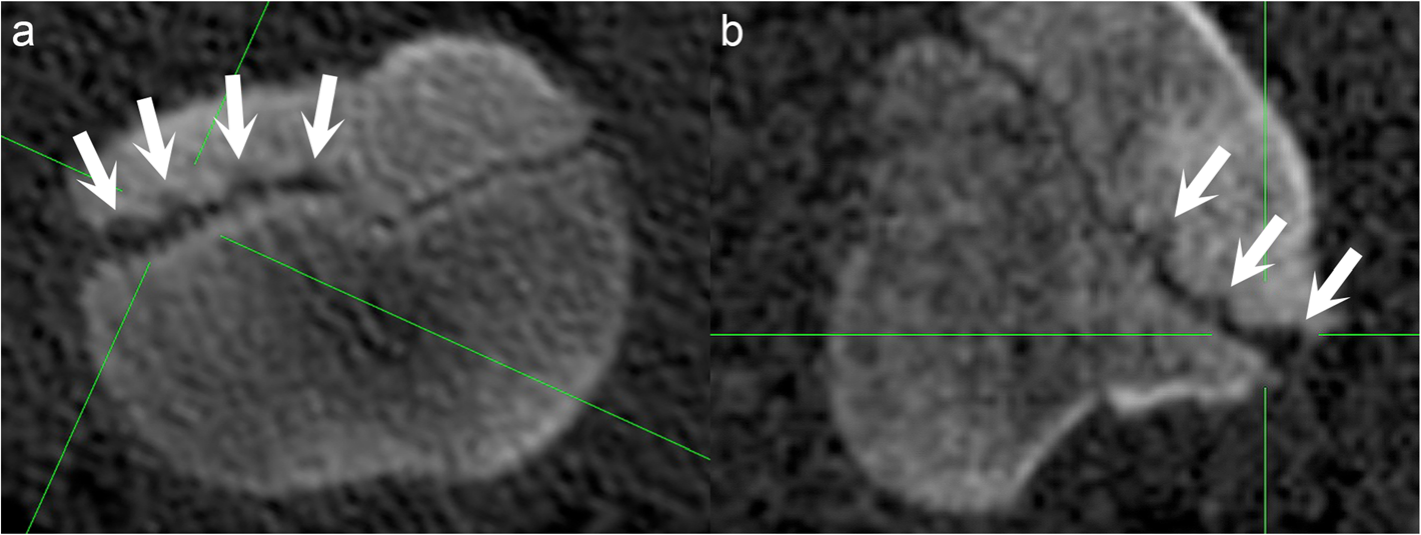
Multi-lobulated, stair-step lesions. Proximal is to the top and distal is to the bottom of each image. **a** Medial is to the left and lateral is to the right of the image. **b** Cranial is to the left and caudal is to the right of the image. **a** Pig 5, interval 1, left distal femur, frontal plane. There is a multi-lobulated lesion in the metaphyseal-side ossification front, consisting of four triangles close together (arrows) referred to as a stair-step lesion. **b** Pig 5, interval 1, left distal femur, sagittal plane. The lesion in **a** also contains three triangles close together in the sagittal plane (arrows)

Lesions occurred in the metaphyseal-side ossification front, epiphyseal-side ossification front, or both. Metaphyseal-side defects were most common. Lobes of metaphyseal-side defects tended to be triangular, but rectangular and semi-circular lobes were also observed. Epiphyseal-side defects tended to be semi-circular. Single defects were observed, but lesions were often multi-lobulated. The most common multi-lobulated geometry was 2–3 closely adjacent, same or different depth triangles (stair-step lesions; Fig. 3), but combinations of rectangles and triangles and up to 5 triangles in single frontal CT slices were sometimes seen.

**Fig. 4:**
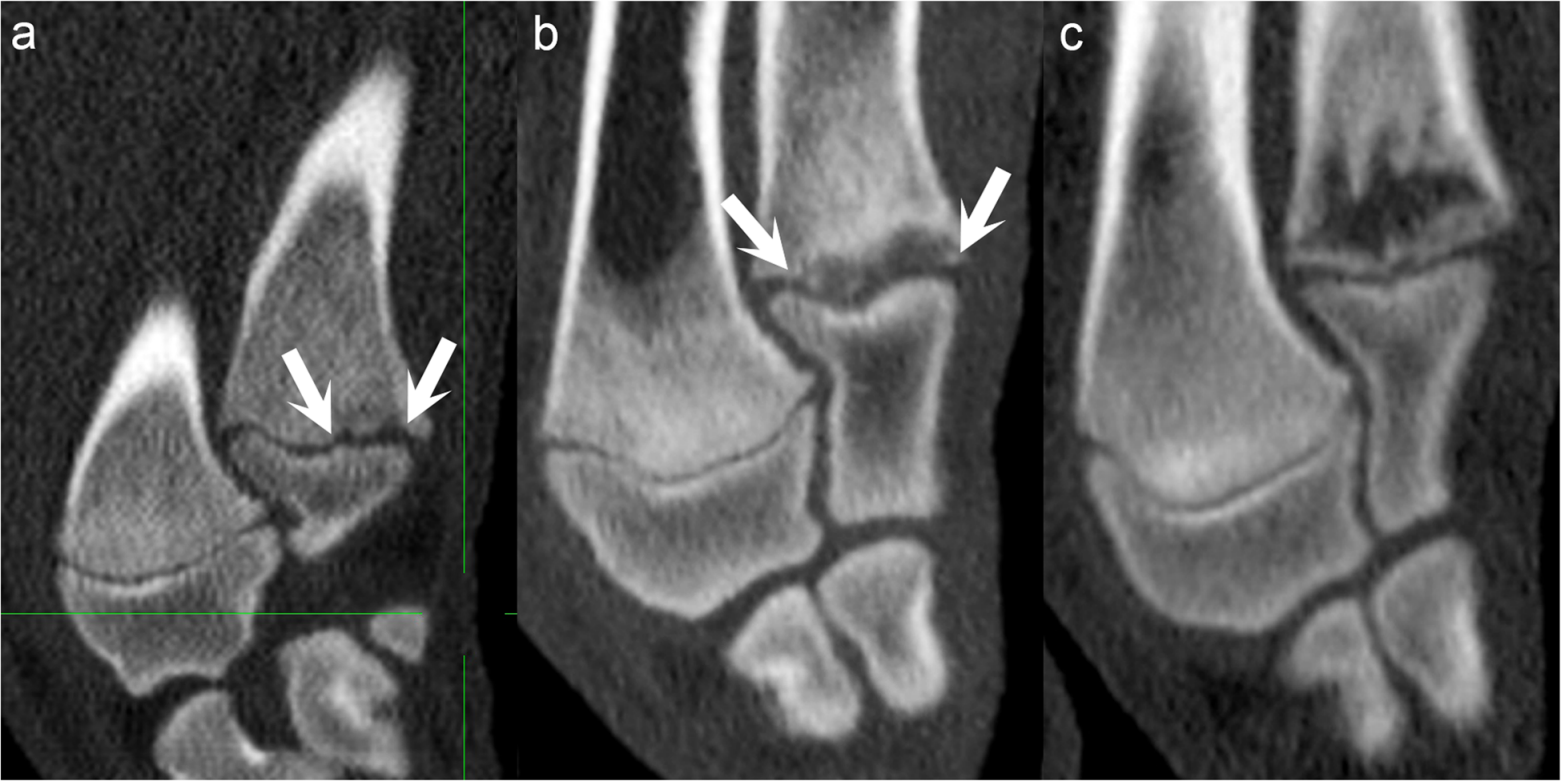
Lesion size and development.All images are in the sagittal plane. Proximal is to the top, distal is to the bottom, cranial is to the left and caudal is to the right of each image. **a** Pig 15, interval 7, right distal ulna. There is a multi-lobulated lesion (arrows) that affects ≤25% of the physis. **b** Pig 10, interval 6, left distal ulna. There is a rectangular lesion (arrows) that affects ~ 75% of the physis. **c** Pig 10, interval 7, left distal ulna, sagittal plane. Continued ossification has caused the lesion in (**b**) to increase in size and move away from the physis, towards the diaphysis

Individual triangles were typically 1–2 mm wide and 2–4 mm deep, rectangles were 2–3 times larger and multi-lobulated lesions tended to be limited to ≤25% of the total size of an affected physis (Fig. 4a). As a notable exception, lesions that were much larger, i.e. spanned 50–75% of the physis occurred in 17 physes of nine pigs, limited to the distal ulnar (Fig. 4b) and distal fibular physes during intervals 4–8. In 13 cases, the suddenly-large defects occurred at the site of previous small defects, and in 4 cases they occurred at the time of first detection. With increasing duration, continued ossification caused metaphyseal defects to appear to gradually increase in size and to move away from the physis, towards the diaphysis (Fig. 4c).

**Fig. 5:**
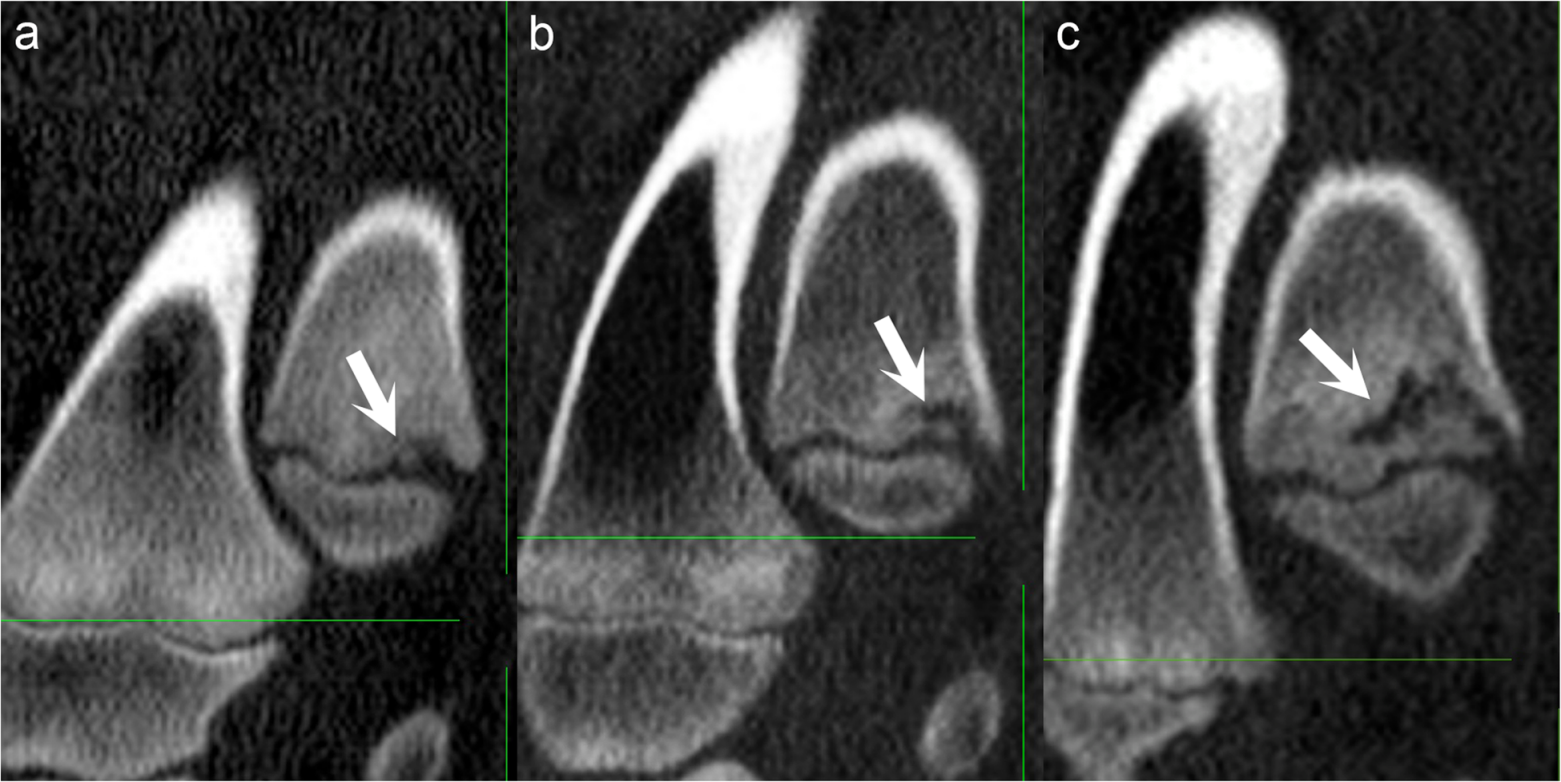
Secondary reparative ossification. All images are in the sagittal plane. Proximal is to the top, distal is to the bottom, cranial is to the left and caudal is to the right of each image. **a** Pig 16, interval 6, left distal ulna. There is a mineral hyperdense focus (arrow), interpreted as a separate centre of reparative ossification, at the transition between the lesion and the physis. **b** Pig 18, interval 4, left distal ulna. There is a linear hyperdensity (arrow) that is connected with the metaphyseal-side ossification front. **c** Pig 14, interval 7, left distal ulna. There is a linear hyperdensity (arrow) that extends horizontally into the defect, causing it to appear reverse-C-shaped

Secondary responses to the primary hypodense defects developed, detectable as three different categories of bone formation. The first category was sclerosis on the margin of lesions towards bone. The second category was formation of mineral hyperdense foci, interpreted as separate centres of reparative ossification, that tended to appear at the transition between the defect and the physis (Fig. 5a). The hyperdense focus often became connected with the metaphyseal-side ossification front (Fig. 5b) and extended horizontally into triangular or rectangular defects, causing them to appear reverse-C-shaped (Fig. 5c). The hyperdense focus then frequently proceeded to form a complete horizontal line parallel with the physis. On some occasions, a second horizontal line developed next to the first. Ultimately, reparative ossification caused defects to fill with bone and resolve.

**Fig. 6:**
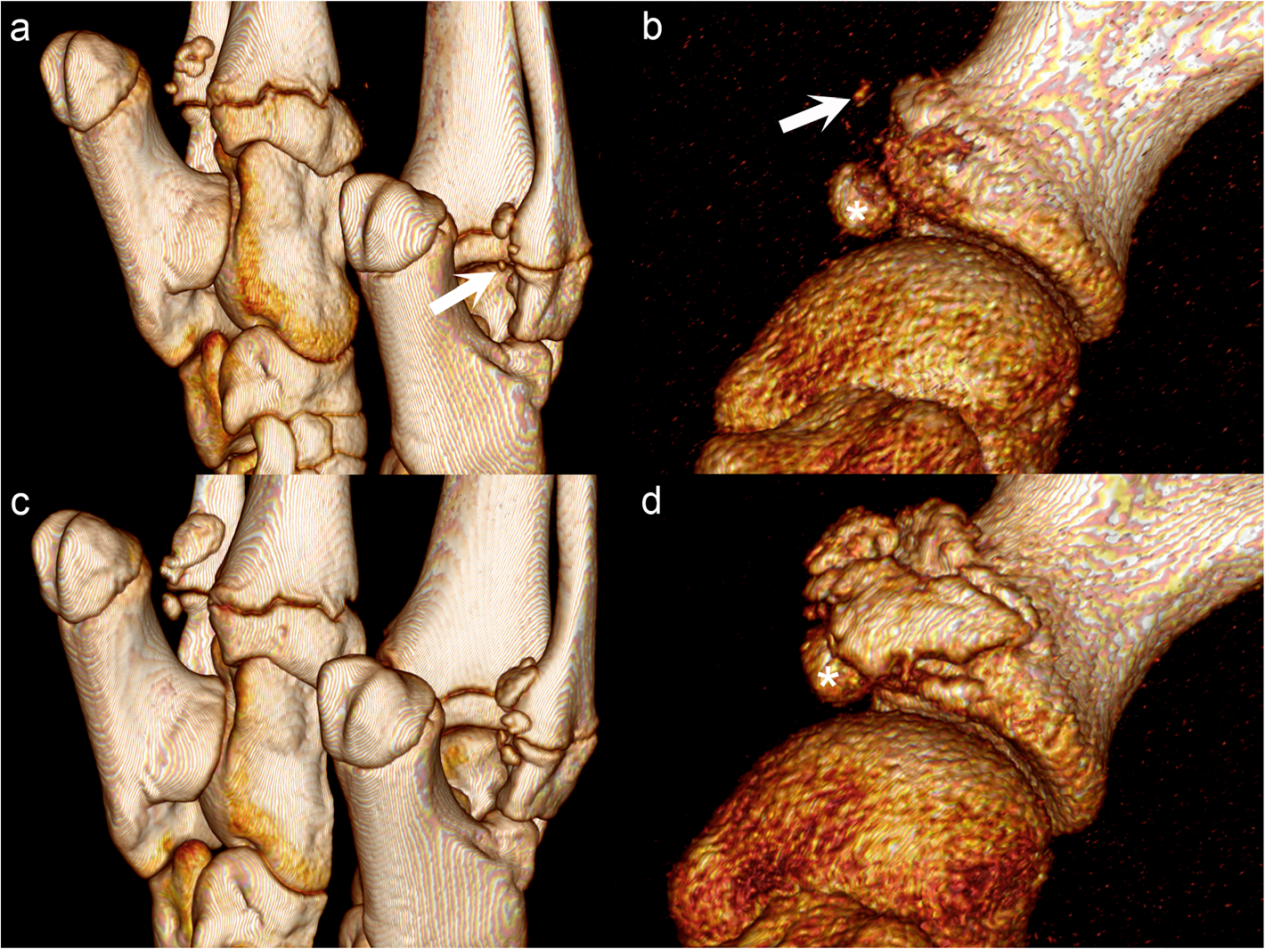
Perichondrial new bone formation. **a** and **c** are caudo-lateral views, **b** and **d** are lateral views. **a** Pig 16, interval 6, left and right tarsi. There are tiny, mineral hyperdense foci (arrow) at the level of, but peripherally adjacent to the right distal fibular physis, i.e. in the location of the perichondrium. **b** Pig 14, interval 7, left shoulder. There is a tiny, mineral hyperdense focus (arrow) adjacent to the right distal scapular physis. **c** Pig 16, interval 7. The foci from (**a**) have increased in size and resemble osteochondrosis dissecans fragments next to the physis, as opposed to in the joint space. **d** Pig 14, interval 8. Some of the mineral hyperdense foci from (**b**) have fused with the underlying bone and form large, florid protrusions. Asterisks (*) in (**b**) and (**d**): Normal ossification centre for the supra-glenoid tubercle

The third category of bone formation was perichondrial new bone, which formed in 11 physes of seven pigs, limited to the distal fibular (*n* = 6) and distal scapular (*n* = 5) physes during intervals 5–8. Tiny, mineral hyperdense foci appeared at the level of, but peripherally adjacent to the physis, i.e. in the location of the perichondrium (Fig. 6a–b). The foci increased in size until they for a time resembled OCD fragments adjacent to the physis (Fig. 6c), as opposed to in the joint space. The fragments then coalesced and fused with the bone they formed next to, after which time they constituted large, florid protrusions (Fig. 6d). The protrusions underwent some remodelling and smoothing, but by the end of the study, bones with perichondrial new bone formation were deformed in terms of having grossly enlarged physeal regions compared to lesion-free counterparts. The distal fibular and distal scapular physes were therefore potentially qualitatively more important than other physes because lesions were associated with gross deformity.

## Discussion

The development of physeal osteochondrosis in pigs was followed longitudinally by CT. The probably most important result was new information about pathogenesis.

### The main trigger for physeal and articular osteochondrosis is vessel incorporation into bone

The current results represent the longitudinal data from a project during which lesions in the right distal femoral physis of the included pigs were previously histologically validated [9]. In histological sections, lesions were centred on necrotic cartilage canal vessels and interpreted to represent vascular failure [9]. All distal femoral physeal lesions were multi-lobulated or multiple, and the only anatomical structure that matched the distribution of the multiple lobes was the blood supply [9, 14]. The blood supply is organised as end arterial trees, with a trunk and smaller branches (Fig. 12 in [21]). Failure of a vessel trunk at the point of incorporation into bone is associated with devascularisation of cartilage around multiple, smaller branches simultaneously, and the devascularised areas form the lobes of multi-lobulated lesions (Figs. 10–12 in [21]). During longitudinal study of articular osteochondrosis, there were 1–2 peaks in the incidence curves for the examined joints [8]. In the absence of any other predisposing factor common to several pigs at the same time, it was hypothesised that most pigs have 1–2 vessel trunks left to incorporate into bone at similar times up to 180 days (8, Figs. 13–14 in [21]). The probably most important finding of the current study was that there were 1–2 peaks in the incidence curves for most examined physes, as this corroborates that vascular failure occurs by the same mechanism in physeal and articular osteochondrosis. If the mechanism is the same, the predisposing genes may also be similar.

### Variable physis prevalence may be due to duration and relative contribution to growth

There were only minor differences in the prevalence of lesions between physis in the current, 1978 [3] and 1984 [10] generations of pigs (Table 4). One of the main challenges in osteochondrosis research is to explain why lesions are common at predilection sites in some physes and epiphyses, whereas others are rarely affected [3, 11, 17]. Variable physeal prevalence could theoretically be explained by having different numbers of vessels to incorporate into bone, but we find it hard to believe that basic physiology should be that different and think it more likely that the explanation is to be found among disease-moderating, than among disease-initiating factors. Reiland reviewed proposed explanatory factors of overloading, instability and unfavourable shape, before stating that local growth potential was the factor he felt was most likely to have the greatest and most primary influence on lesion distribution [3]. In epiphyseal growth cartilage, there is topographical variation in the age of physiological regression of the blood supply [5, 6, 23–25], and predilection sites for OCD/osteochondral fragmentation in foals were the last to lose their blood supply in several bones [5, 24, 25]. Similarly, it is well-documented that different physes close at different ages, and the physes with the most lesions corresponded to the physes with the longest closing times in the fore- and hind limbs, respectively (Table 1, [19, 20]). Duration of growth may therefore be part of the explanation for why lesions are more common in some physes than in others. The opposite was not true: the physes with the fewest lesions did not correspond to the physes with the shortest closing times [19, 20]. However, it is also well-known that proximal and distal physes contribute differentially to growth [22], and there was a perfect match between which physis contributed more to growth and which physis had more lesions out of proximal and distal physes in 5/6 examined bones, the exception being the fibula (Table 4). The current results therefore seem to suggest the exact same thing as Reiland did 40 years ago [3]: the reason that lesions are more common in some physes than others is due to a combination of duration and relative contribution to growth. Greater contribution could entail greater susceptibility to the deleterious effects of premature devascularisation.

**Table 4:** Literature on relative prevalence of osteochondrosis between physes

Study	Reiland, 1978 [3]	Hill et al., 1984 [10]	Hill et al., 1985 [11]		Current results	Order of closing according to Zeder et al., 2015 review [19]
	176 pigs	60 pigs	32 pigs		18 pigs	
Breed	Swedish Landrace, Yorkshire and mixed	Purebred and crossbred	Crossbred		Norwegian Landrace	
Age	3–18 months	25–169 days	1 day-old	15 day-old	70–180 days	
Ranked by	Frequency and severity	Frequency	Frequency	Frequency	Frequency at start + developed during study	
Rank	1. Distal ulna 2. Distal femur 3. Costochondral junctions 4. Femoral head 5. Humeral head 6. Ischiatic tuberosity 7.Thoraco-lumbar vertebrae	1. Proximal femur 2. Proximal humerus 3. Distal femur 4. Distal humerus 5. Rib 8 6. Distal ulna 7. Proximal tibia 8. Distal radius 9. Rib 7 10. Distal tibia 11. Proximal ulna 12. Rib 6 13. Rib 5 14. Ischial tuberosity 15. Distal fibula and proximal radius	1. Proximal humerus 2. Proximal tibia 3. Distal femur 4. Distal ulna 5. Distal radius and proximal femur 6. Distal humerus, proximal radius, proximal ulna, distal tibia and ischial tuberosity all 0 lesions	1. Proximal and distal humerus 2. Distal femur 3. Proximal tibia 4. Proximal femur 5. Distal radius 6. Proximal radius, proximal and distal ulna, distal tibia and ischial tuberosity all 0 lesions	1. Distal femur 2. Distal ulna 3. Proximal humerus 4. Proximal tibia 5. Proximal femur 6. Distal fibula 7. Distal humerus 8. Distal scapula, distal radius, distal tibia 9. Proximal radius 10. Proximal fibula 11. Proximal ulna	1. Axis 2. Atlas 3. Pelvis 4. Scapula 5. Proximal radius 6. 2nd phalanx 7. Distal humerus 8. 1st phalanx 9. Distal tibia 10. Metatarsal 11. Metacarpal 12. Distal fibula 13. Calcaneum 14. Proximal femur 15. Distal radius 16. Proximal ulna 17. Distal femur 18. Proximal tibia 19. Distal ulna 20. Proximal fibula 21. Proximal humerus
Lesions more common in proximal or distal physis						Relative contribution to growth according to Payton, 1932 [22]
Humerus	N/a	Proximal > distal	Proximal > distal	Proximal = distal	Proximal > distal	Proximal > distal
Radius	N/a	Distal > proximal	Distal > proximal	Distal > proximal	Distal > proximal	Distal > proximal
Ulna	Distal > proximal	Distal > proximal	Distal > proximal	0 lesions	Distal > proximal	Distal > proximal
Femur	Distal > proximal	Proximal > distal	Distal > proximal	Distal > proximal	Distal > proximal	Distal > proximal
Tibia	N/a	Proximal > distal	Proximal > distal	Proximal > distal	Proximal > distal	Proximal > distal
Fibula	N/a	N/a	N/a	N/a	Distal > proximal	Proximal > distal

### All physeal lesions are potentially destined to resolve

**Fig. 7:**
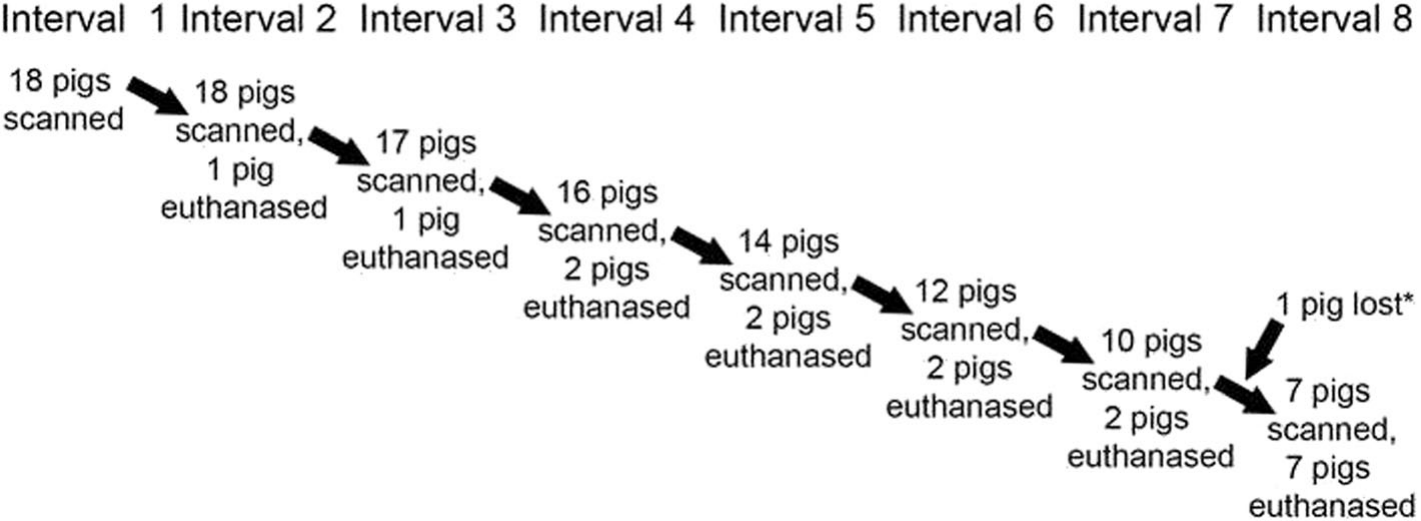
Flowchart of study design. The 18 pigs were examined eight times at biweekly intervals. From the second interval, 1–2 pigs were euthanased for histological validation (reported elsewhere) [9] at each interval. The pig identified as number 10 was euthanased due to acute respiratory distress between intervals 7 and 8 (asterisk*). At the eighth interval, all remaining pigs were euthanased

The median proportion of lesions that resolved was higher, and the range was greater for physeal osteochondrosis than for articular osteochondrosis in these pigs [8]. Hill et al. described that many lesions appeared to reach a certain size and then resolve [17]. On one hand, this fits with vessels being regularly spaced in growth cartilage [26], and the metaphyseal-side ossification front meeting with a second, intact vessel some time after vascular failure, resulting in restoration of required factors and horizontal reparative ossification into lesions (Fig. 7 and [9]) On the other hand, in the previous study, lesion size varied from 1.1–45.6% of physeal width [9], and in the current study, suddenly-large lesions were observed in the distal ulnar and distal fibular physes. Osteochondrosis lesions are only delineated in plain CT scans in the extent to which they are surrounded by mineralized tissue, i.e. advancing ossification, and suddenly-large lesions are likely to be the result of growth spurts. If growth spurts vary between physes, they potentially add to the hypothesis of Reiland that variable physeal prevalence is due to differences in local contribution to growth, discussed above [3].

The observation that 100% of lesions resolved in the proximal ulnar physis and other factors make it plausible that the proportion of lesions that resolve is greater for physeal than for articular osteochondrosis [17]. A second challenge in osteochondrosis research is to explain why some lesions progress to cause clinical signs, whereas others do not. Size, as discussed above, is believed to be part of the explanation for whether articular and physeal lesions resolve or progress to cause clinical signs [9, 27–29]. If most physeal lesions are destined to resolve [17], then the rest of the explanation cannot be that unresolved lesions progress to cause clinical signs (because all lesions resolve). Instead, we propose that relative duration of lesions from initiation to resolution may play a part. Hill et al. seemed to suggest that duration was relatively constant at 4–6 weeks [17], but the current results show that averaged lesion duration can range from 18 to 67 days depending on physis. Thus, the following hypothesis emerges: lesions that affect 1.1% of physeal width [9] and last for 18 days are unlikely to cause clinical signs, whereas lesions that affect 45.6% of physeal width [9] and last for 67 days are likely to cause clinical signs.

None of the current pigs had any limb deformity. This is not surprising, and does not rule out the association because subclinical lesions are common in 4 month-old pigs, whereas pronounced deviation is more apparent in 6–8 month-old pigs [3]. Lesions dominated in the medial and central/caudal halves of the distal femoral physis (72%, [9]), corresponding to medial shortening and caudal rotation seen in the femurs of 8 and 9 month-old pigs in Figs. 1 and 2 of Reiland (1978) [3]. Angulation is probably due to retained chondrocytes constituting an obstacle to osteoblast advancement, or reparative ossification resulting in premature bone bridging [3, 9], but the exact mechanism will have to be determined by examination of pigs older than 6 months [3].

Lesions in the distal scapula and distal fibula were associated with perichondrial new bone formation that led to permanent physeal enlargement. Bittegeko & Arnbjerg noted physeal enlargement in the distal ulna [18]; this was not one of the bones with perichondrial new bone formation in the current study; perhaps it occurs there, just not in this study, or perhaps it occurs as a late effect of the other two categories of reparative ossification. Radiologically, the lesions with perichondrial new bone formation were reminiscent of how rib fractures heal with excessive callus due to continued respiratory movement, thus healing osteochondrosis lesions may be subject to different biomechanical conditions in some physes. Decubital ulcers commonly develop at bony protrusions with minimal soft tissue covering [30]; enlarged bones could exacerbate this problem and this further emphasizes the importance of selecting against physeal osteochondrosis. The florid stage of perichondrial new bone formation was potentially radiologically compatible with septic physitis/osteomyelitis. Bacteria can bind in cartilage canals and cause vascular failure [31–33], and because this affects the same anatomical component: the cartilage canals, lesions can have the exact same geometry and distribution as heritably predisposed, aseptic vascular failure [34]. Currently, the only way to differentiate septic from aseptic vascular failure is through identification of bacteria and perivascular neutrophils within canals in histological sections [33]. However, bacteria are responsible for a minority of lesions [34–36], and perivascular neutrophils were only found in pig 5 of the current study [9]. Pig 5 was not one of the pigs with florid lesions, and the florid lesions in this study were therefore associated with aseptic, as opposed to septic vascular failure.

### Limitations

Assessment of angular limb deformity could have been improved by using more objective methods. Subtle cases may have been missed, leading to underestimation of the true relationship between physeal osteochondrosis and angular limb deformity. Likewise, CT scans read by a single observer have lower reliability than scans read by multiple observers. Agreement was higher at 81% for physeal lesions, compared to 71.5% previously reported for evaluation of articular lesions by the same observer [8]. This probably reflects increased experience, but may also partly reflect the subjective impression that physeal lesions were easier to read by virtue of being located between two ossification fronts, as opposed to superficial to a single ossification front for articular lesions. It was not financially viable to use more than one observer. However, we are continuously working towards developing more objective, automated methods for evaluation of conformation and CT scans that may reduce these limitations in future.

Computed tomography can be used for phenotyping of physeal osteochondrosis, and could influence the outcome of selection as described for pigs 18, 13 and 15. The potential for false-negative diagnoses due to lesions having resolved before 120 kg body weight has been quantified. It appeared that the windows for lesions arising had not closed [8, 15, 16], meaning there was greater potential for false-negative diagnoses due to lesions not having arisen yet at 120 kg than previously documented for articular lesions in the stifle and elbow joints (2%, [8]). The time point for scanning is unlikely to be moved because quantification of lean meat and fat percentage is standardised at this weight, and because most pigs finish at approximately this live weight. The CT table function is guaranteed up to 200 kg, thus the upper age limits for lesions arising could be investigated further in future. Phenotyping is unlikely to go out of fashion even if genomic selection supervenes, because it is necessary to monitor the effects of selection and confirm associations at regular intervals.

## Conclusions

Physeal osteochondrosis followed a similar dynamic development pattern to articular osteochondrosis. There were peaks in the incidence curves, compatible with intermittent failure of cartilage canal vessels during incorporation into bone. In some physes, osteochondrosis led to permanent enlargement, potentially relevant to decubital ulcers. The relationship between physeal osteochondrosis and angular limb deformity must be examined further in pigs over 6 months old in future.

## Methods

The study was approved by the National Animal Research Authority (FOTS ID: 2010/2630). All pigs were kept in accordance with the national legislation (Animal Welfare Act LOV-2009-06-19-97; Regulation for the keeping of pigs in Norway FOR-2003-02-18-175).

The material consisted of 18 male Landrace pigs that were part of a previous project on articular osteochondrosis [8, 14]. The pigs were purchased from members of the Norwegian pig breeders’ association, Norsvin SA (www.norsvin.no), with informed consent for the pigs to enter research. The pigs were sired by a boar with high breeding value for articular osteochondrosis, and were the offspring of eight sows whose farrowing dates fitted with the study start date. The pigs were from 70 to 82 days old and weighed from 20 to 38 kg (mean: 26 kg) on the study start date. The 18 pigs were examined eight times at biweekly intervals (Fig. 7). The pigs were visually inspected for angular limb deformity from in front and behind by a veterinary surgeon (KO) and a food scientist (JK) at each interval. At the first interval, all pigs were anaesthetised and CT-scanned. From the second interval, 1–2 pigs were euthanased for histological validation (reported elsewhere) [9] and CT-scanned, whereas the rest of the pigs were anaesthetised and CT-scanned (Fig. 7). At the eighth interval, all remaining pigs were euthanased and CT-scanned (Fig. 7). Pigs were assigned numbers from 1 to 18 by ascending age on the study end date for each pig, ranging from 96 to 180 days, and by ascending body weight if age was equal, ranging from 47 to 128 kg (Table 5). There were 112 available serial CT scans, ranging from 2 to 8 scans per pig (median: 7 scans; Table 5).

**Table 5:**
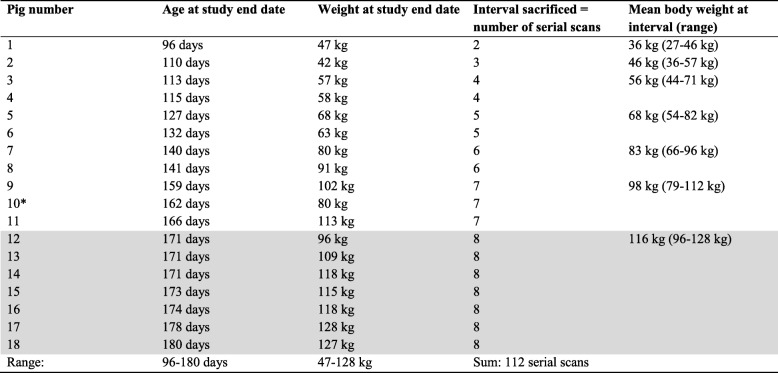
Pigs

*Pig 10 was euthanased due to acute respiratory distress between intervals 7 and 8. Grey shading: The seven pigs that completed all eight intervals

### Immobilisation

At the first and second intervals, pigs were anaesthetised by intra-muscular injection of 2 mg/kg midazolam (Midazolam B, Braun, Melsungen, Germany) and 10 mg/kg ketamine (Ketalar, Pfizer Inc., New York, USA). From the third interval, anaesthesia was changed to 2.2 mg/kg xylazine (Rompun vet, Bayer Animal Health GmbH, Leverkusen, Germany) and 4 mg/kg tiletamine/zolazepam (Zoletil Forte Vet, Virbac, Carros, France) to minimise injection volume. Euthanasia was by captive bolt stunning and exsanguination.

### CT scanning

Pigs were positioned in sternal recumbency with the fore- and hindlimbs extended in a 32-slice helical CT (GE Light Speed Pro 32, GE Healthcare, Little Chalfont, UK) at the Norsvin Delta boar test station in Hamar, Norway. After acquisition of a scout image, scan windows were collimated to acquire a series of transverse slices from the front claws to the top of the scapula, and from the tuber coxae to the hind claws of each pig, using a fixed kV of 120, dynamic mAs up to 650 and a slice thickness of 0.625 mm.

### Evaluation of CT scans

Scans were imported into a picture archiving and communication system for evaluation (www.osirix-viewer.com) and scored by a single, veterinary radiologist observer (KO). Seven forelimb physes and six hind limb physes were evaluated, comprising the distal scapular physis and the proximal and distal physes of the humerus, radius, ulna, femur, tibia and fibula. The 13 physes were evaluated in the left and right limbs of the pig, i.e. 26 physes were evaluated in each CT scan.

**Fig. 8:**
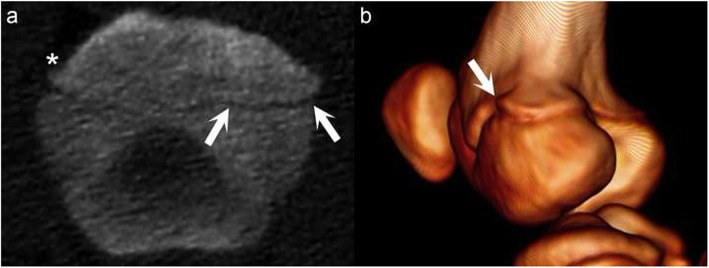
Changes considered representative of normal growth. Proximal is to the top and distal is to the bottom of each image. **a** Medial is to the right and lateral is to the left of the image. **b** Caudo-medial view. **a** Pig 2, interval 3, right distal femur, frontal plane. The physis has an undulating profile, free from focal widening. There is gradual widening of the physis towards the periphery medially (between arrows) and laterally. A narrow rim of bone extending slightly beyond the periphery of the remainder of the bone is visible laterally (below asterisk*; “lipping”). **b** Pig 2, interval 3, right stifle. There is a narrow rim of bone (arrow) extending slightly beyond the periphery of the remainder of the bone, towards part of the margin of the physis (“lipping”)

The following changes were considered representative of normal growth and not counted as lesions: a profile that was undulating, but free from focal widening (Fig. 8a); gradual and/or diffuse irregularities (Fig. 8a), and a narrow rim of bone extending slightly beyond the periphery of the remainder of the bone, towards part or the entire margin of a physis (“lipping”; Fig. 8).

Based on previous studies, osteochondrosis lesions were defined as focal, sharply demarcated areas of soft tissue hypodensity in or near the epiphyseal-side or metaphyseal-side ossification fronts of the physis [9, 14]. The definition included single defects and multiple defects close together, referred to as multi-lobulated lesions. The number of lobes within multi-lobulated lesions and exact location of lesions within a physis were not recorded, but rather each physis was assigned an overall score of lesion-negative or lesion-positive at each interval. There were three subcategories within each of the two main scores: lesion-negative physes were negative at the study start, negative because a lesion from the preceding interval resolved or negative without having been positive at the preceding interval. Lesion-positive physes were positive at the study start, positive because a lesion developed during the study or positive because a lesion persisted from the preceding interval.

### Agreement

Agreement was informally assessed by scoring all 18 pigs on the study end date on two occasions 6 months apart. This generated 468 paired observations with agreement on 378 occasions (81%).

### Articular osteochondrosis

All raw data from the previous studies of articular osteochondrosis in these pigs were available [8, 14], and were used to test correlation between the number of physeal and articular lesions.

## Data Availability

The datasets used and analysed in the study are available from the corresponding author on reasonable request.

## Abbreviations

CT: Computed tomography
OCD: Osteochondrosis dissecans
